# Insights into ALS pathomechanisms: from flies to humans

**DOI:** 10.1080/19336934.2015.1114694

**Published:** 2015-11-23

**Authors:** Andrea Chai, Giuseppa Pennetta

**Affiliations:** 1 Euan McDonald Center for Motor Neurone Disease Research; 2 Centre for Integrative Physiology; University of Edinburgh; Edinburgh; UK

**Keywords:** Amyotrophic Lateral Sclerosis, cell proliferation/apoptosis, computational analysis, *Drosophila* model, endocytosis, human tissue validation, large scale modifier screen, lipid droplets, VAMP-Associated Protein B

## Abstract

Amyotrophic Lateral Sclerosis (ALS) is a devastating neurodegenerative disease causing the death of motor neurons with consequent muscle atrophy and paralysis. Several neurodegenerative diseases have been modeled in *Drosophila* and genetic studies on this model organism led to the elucidation of crucial aspects of disease mechanisms. ALS, however, has lagged somewhat behind possibly because of the lack of a suitable genetic model. We were the first to develop a fly model for ALS and over the last few years, we have implemented and used this model for a large scale, unbiased modifier screen. We also report an extensive bioinformatic analysis of the genetic modifiers and we show that most of them are associated in a network of interacting genes controlling known as well as novel cellular processes involved in ALS pathogenesis. A similar analysis for the human homologues of the *Drosophila* modifiers and the validation of a subset of them in human tissues confirm and expand the significance of the data for the human disease. Finally, we analyze a possible application of the model in the process of therapeutic discovery in ALS and we discuss the importance of novel “non-obvious” models for the disease.

## Introduction

Amyotrophic Lateral Sclerosis (ALS) is a fairly common neurodegenerative disease characterized by the selective death of motor neurons and a progressive decline in muscle function leading to paralysis, speech deficits and eventually death due to respiratory failure.^1^ ALS was first described more than 130 years ago by the French neurologist Jean-Martin Charcot and yet, the understanding of the molecular mechanism underlying disease pathogenesis remains elusive.^2,3^ The disease usually appears in midlife and causes death within 3 to 5 years after clinical onset.^1^

Approximately 10% of ALS cases are inherited and are classified as familial while the majority are sporadic with no apparent genetic cause. Familial and sporadic ALS cases share common pathological features leading to the hypothesis that studying the mechanism of disease pathogenesis for the familial cases will provide relevant information for the more common sporadic cases.^1^

In 1993, missense mutations in the gene encoding the Cu/Zn superoxide dismutase 1 (SOD1) in a subset of ALS familial cases,^4^ led to the conviction that finding a therapy for this devastating disease was going to be a relatively simple task. Since the normal function of SOD1 is the conversion of superoxide anions into hydrogen peroxide, it was initially thought that a decrease in its catalytic activity with a consequent accumulation of free radicals is responsible for the toxic effect associated with ALS-causing SOD1 alleles. However, subsequent studies focusing on the elucidation of the mechanism underlying SOD1-mediated toxicity revealed that understanding this mechanism was unexpectedly challenging. Several lines of transgenic mice expressing various ALS-causing SOD1 variants were generated and they were found to recapitulate major hallmarks of the human disease.^5^ However, studies on these murine models showed that various SOD1 mutations exhibit a remarkably high degree of variability in their enzymatic activity and more importantly, there is no direct correlation between the degree of this activity on one hand and the onset and severity of the disease on the other hand.^5^ These data together with the finding that SOD1 knock-out mice do not develop motor neurone diseases led to the conclusion that ALS-causing SOD1 variants are not loss-of-function alleles but rather neo-morphs that have acquired new toxic properties.^5^

Initial attempts to generate *Drosophila* models for SOD1-induced ALS, provided disappointing results as well.^6^ Expression of *Drosophila* Sod gene in a number of tissues has little or no effect on life-span while expression of its human homolog in flies increases life-span more than 40%.^7^ In contrast to mice knockout for SOD1, flies deficient for the same gene exhibit early lethality that can be rescued by the targeted expression of the SOD1 human gene in motor neurons.^8^ Additionally, high levels of expression of ALS causing SOD1 alleles in *Drosophila* motor neurons have no deleterious effects but instead induce a significant extension in fly life-span and rescue the mutant phenotype due to the inactivation of the fly endogenous gene.^9^ Hence, the toxic effect associated with the expression of ALS causing SOD1 alleles in mice and in humans is not observed in *Drosophila*.

A more recent study showed that motor neuron specific expression of wild-type or disease-linked mutants of human SOD1 in *Drosophila* does not affect life-span but instead induces a progressive decline in locomotion activity that is associated with defective neuronal physiology and induction of stress response in surrounding glia cells. However, these phenotypes were not accompanied by a decrease in neuronal viability despite the fact that neurons exhibit a severe accumulation of mutant SOD1-induced aggregates.^10^ Recently, another paper has been published reporting that transgenic flies expressing a Zn-deficient SOD1 allele display a progressive decline in locomotion performance and in their muscles accumulation of abnormal mitochondria and reduced ATP production are observed. However, these flies do not exhibit any neuronal degeneration and their life-span is normal.^11^

In conclusion, many transgenic SOD1 mice mimicking the human disease are available and several attempts have been made to generate a fly model for this type of ALS, nevertheless, the mechanism of SOD1-mediated toxicity remains an open question.

In 2004 more than 10 y after the identification of SOD1 as an ALS causative gene, a missense mutation in a conserved region of the human VAMP/Associated Protein B (hVAPB) was shown to cause a range of motor neuron diseases including spinal muscular atrophy, atypical ALS and typical ALS (ALS8).^12^ The ALS causative mutation replaces the Proline in position 56 with a Serine (P56S) and this amino acid residue is conserved from yeast to man. We and other labs generated a first *Drosophila* model for ALS8 by expressing the *Drosophila* protein carrying the ALS8 causing mutation (*DVAP-P58S*) in flies. Expression of the disease transgene in neurons recapitulates major features of the human disease including aggregate formation, neurodegeneration, locomotion defects and decreased life-span.^13-16^ Recently, 2 additional missense mutations in hVAPB have been shown to cause ALS8 in humans. Expression of these mutant transgenes in flies also reproduces hallmarks of the human disease confirming that *Drosophila* is an excellent system in which to model ALS8.^17,18^

Loss of *DVAP* function results in structural changes at the larval neuromuscular junction (NMJ) that are characterized by a decrease in the number of synaptic boutons and an increase in their size while presynaptic overexpression of the same protein leads to an increase in the number of boutons and a decrease in their size.^19^ These morphological changes are accompanied by an increase in the number of active zones in the *DVAP* mutants and a decrease of vesicle density in *DVAP* overexpressing synapses. Despite the observed structural remodelling at the larval NMJ, the synaptic response following nerve stimulation remains the same in both genotypes and is similar to the wild-type. However, quantal size was found to be increased in *DVAP* mutants and decreased in *DVAP* overexpressing synapses suggesting that compensatory changes in quantal size may be responsible for maintaining synaptic response within functional boundaries. In agreement with these changes in synaptic physiology, DVAP expression levels affect the abundance of specific subunits of postsynaptic glutamate receptors and the volume of the postsynaptic receptor clusters.^13^

Interestingly, similar alterations in synaptic morphology are associated with the expression of the human protein hVAPB in fly neurons and more importantly, expression of hVAPB in a mutant background for DVAP rescues the synaptic phenotypes and the lethality associated with DVAP mutants.^13^ Taken together, these data clearly show that the human and *Drosophila* proteins are functionally interchangeable and that information gained by studying the function of DVAP can be relevant to the function of its human homolog. Moreover, the N-terminal fragment of VAP proteins encoding the major sperm protein (MSP) domain, has been shown to be secreted and to control mitochondria structure and function in muscles by interacting with a number of axon guidance receptors such as Ephrin receptors, Robo and Lar-like protein tyrosine phosphatases.^14,20^

Interacting DVAP proteins have been recently identified and they include the oxysterol binding proteins and the Down syndrome cell adhesion molecule Dscam.^21,22^ DVAP protein interacts with the cell-adhesion molecule Dscam and determines its localization at the axonal processes where Dscam affects axonal branching by controlling self-recognition and avoidance processes between neurons.^22^ Finally, DVAP has been shown to co-localize with the phosphoinositide phosphatase Sac1 (Suppressor of Actin 1) to control phosphoinositide levels in neurons. Moreover, expression of the ALS8 allele in neurons sequesters Sac1 into the aggregates leading to its inactivation. The consequent increase in phosphoinositide levels induces structural changes at the NMJ and neurodegeneration. Notably, inactivation of the corresponding phosphoinositide kinase in neurons decreases phosphoinositide levels and suppresses DVAP-P58S neurodegenerative phenotype in the eye. Taken together, these data show that increase in phospoinositide levels plays a crucial role in ALS pathogenesis and identify phosphoinositide kinases as a possible target for therapeutic intervention.^16^

Over the past 5 years, a number of additional genes have been shown to cause ALS and the vast majority of them can be clustered into 2 functional categories: genes affecting RNA metabolism such as TDP43 and FUS and genes that affect protein degradation by controlling the 2 major protein clearance pathways: the autophagy and the ubiquitin/proteasome system.^23^ VAP proteins have been shown to participate in protein clearance by controlling the unfolded protein response that appears to be defective when the ALS8-linked mutation is expressed.^17,24^ Studies in *Drosophila* and mammalian cells showed that P56S disease variant is a loss of function allele by a dominant negative mechanism as it inactivates the wild-type activity by sequestering the endogenous protein into the cytoplasmic aggregates.^15,16,25^ In line with the proposed disease mechanism, hVAPB levels have been found to be decreased in all sporadic cases investigated^26^ and in ALS mouse models for TDP-43 and SOD1.^27,28^ Moreover, TDP-43 associates with VAPB-positive aggregates in transgenic mice expressing the P56S disease-linked allele and the ALS causative gene FUS binds hVAPB RNA.^29,30^ Collectively, these data indicate that hVAPB plays an important role in ALS pathogenesis; however, the molecular mechanism underlying ALS8 pathogenesis remains poorly understood.

Here we show how systematic and unbiased survey of the *Drosophila* genome to search for genetic modifiers of the ALS8 phenotypes can lead to the discovery of important and possibly, novel genes involved in disease pathogenesis. We also show that by combining *Drosophila* genetics and computational approaches of network analyses with subsequent validation of selected modifiers in human tissues, fundamental and conserved pathways underlying ALS pathogenesis can be revealed and elucidated. Finally, we discuss how the use of a *Drosophila* model can assist in the identification of pharmacological targets for therapeutic intervention and the importance of searching for novel and non-obvious models of diseases.

## Generation of the ALS8 Fly Model and Screening Strategy

One way to identify missing components within a genetic network is to conduct a screen for enhancers and suppressors of specific phenotypes. Although it is true that any screen will perhaps identify enough genes to keep a laboratory busy for many years, performing a screen and characterizing the identified hits require an enormous amount of work. Therefore, it is important to spend some time in designing a rapid and effective screening strategy. A preliminary and key consideration is to generate a *Drosophila* model and to determine the most suitable phenotypic readout for the modifier screen. The eye is by far the most popular place in which to conduct such screens because it is easy to score, it is not essential for viability and more importantly, it has been shown to represent an excellent phenotypic readout in successful modifier screens using *Drosophila* models of neurodegenerative diseases.^31^ Moreover, it is in general quite difficult to determine *a priori* whether a screening strategy is going to be successful or not and therefore it is important to test the sensitivity of the disease model in detecting genetic modifiers by performing a quick pilot screen. Bearing this in mind, we found that expression of the *UAS-DVAP-P58S* transgene in the eye by using the *eyeless-GAL4* driver induces a robust eye phenotype characterized by reduced size, fused ommatidia, missing and occasionally, extra bristles.^16^ As a tester line for the screen we selected a transgenic *DVAP-P58S* line that displays a reduction of the eye size to approximately 30% of the wild-type value when flies containing one copy of the transgene are raised at 30°C. A similar phenotype is associated with the expression of a double copy of the transgene in flies raised at 25°C while flies expressing one copy of *DVAP-P58S* at 28°C have a less severe phenotype. Taken together, these data clearly show that the reduction in eye size is not an experimental artifact caused by the increased temperature but is due the expression of the *DVAP-P58S* transgene and it is dependent on its dosage. Additionally, the decrease in eye size to approximately 30% of the wild-type value represents an excellent quantitative metric of modifying effects.

**Figure 1.**
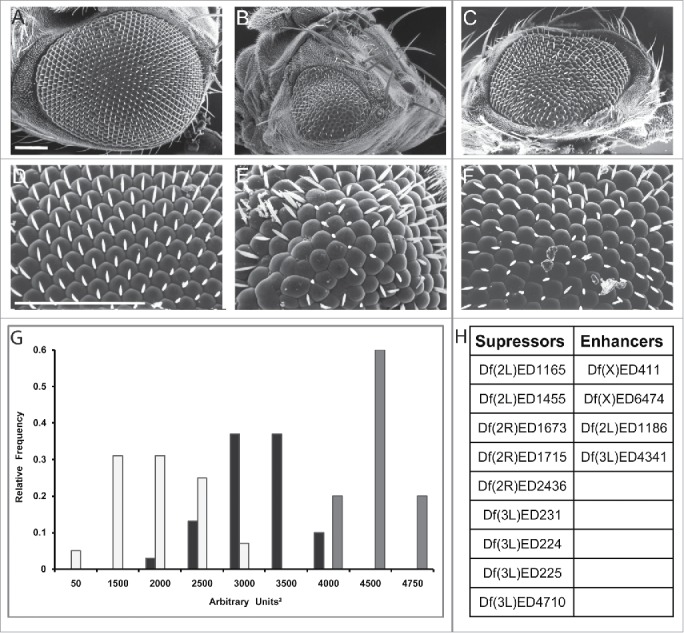
*Df(3L)ED225* suppresses DVAP-P58S-induced eye phenotype. Scanning electron microscopy images of (**A**) *ey-Gal4/+* control flies, (**B**) *ey-Gal4, UAS-DVAP-P58S/+* and (**C**) *ey-Gal4, UAS-DVAP-P58S/+; Df (3L)ED225/+* flies. Flies in (**D**), (**E**) and (**F**) are higher magnifications of the genotypes in (**A**), (**B**) and (**C**) respectively. Scale bars: 100 µm. (**G**) 60% of *ey-Gal4, UAS-DVAP-P58S/+* flies (white) have eye size areas centered between 1500 and 2000 arbitrary square units (N=61, SD=654) and 5% had eye sizes around 50 arbitrary square units compared to 74% of *ey-Gal4, UAS-DVAP-P58S/+; Df(3L)ED225/+* flies (black) that are centered between 3000 and 3500 arbitrary square units and 10% at 4000 arbitrary square units (N=36, SD=378) that is toward control values (60% centered around 4500 arbitrary square units and 20% centered around 4000 and 4750 arbitrary square units respectively. Grey, N=20, SD=261). Differences between eye size distributions of *ey-Gal4, UAS-DVAP-P58S/+* flies and *ey-Gal4, UAS-DVAP-P58S/+; Df(3L)ED225/+*flies were highly significant (P<0.001, according to the non-parametric Mann-Whitney U test when comparing the 2 data sets). Differences between the distributions of eye sizes between *ey-Gal4, UAS-DVAP-P58S/+* and control flies were highly significant (P<0.001, according to the non-parametric Mann-Whitney U test when comparing the 2 data sets). (**H**). Table reporting that the deficiency screen identified 9 genomic deficiencies as suppressors and 4 as an enhancers of the *DVAP-P58S* eye phenotype. Sem experiments were performed as previously described.^16^ Quantification of the eye phenotype was performed using *Oculus v.1*, an in-house developed software.^17^

For simplicity, in the screen we used as a tester line flies expressing one copy of the *DVAP-P58S* transgene raised at 30°C. We screened the *DrosoDel* collection of deficiencies that includes 209 non overlapping deletions of genomic regions^32^ and 9 deficiencies were found to suppress while 5 were found to enhance the eye phenotype (unpublished data, **Fig. 1**). This approach showed that the tester line could identify potential modifiers. However, it turned out that at least for the deficiencies that were tested, the identification of the interacting genes using available P element-induced mutations was quite difficult. This can be due to the fact that hypomorphic mutations in genetically redundant genes may not display any modifying effect or that the modifying effect of genomic deficiencies is due to the simultaneous removal of 2 or more different genes uncovered by the deficiency.^33,34^ Both scenarios may preclude, or at least make the identification of the interacting genes difficult. We therefore decided to screen lines in which the affected gene is overexpressed. Results from this screen have been reported elsewhere.^35^ Here we describe and critically discuss these data. For rapid identification of the modifier genes, we screened a total of 1,136 P-element insertion lines mainly from the EP and the EPgy2 collections. We selected these lines among those that based on the position and orientation of the P-element insertion, were predicted to induce an overexpression of a neighboring gene. Additionally, we selected lines with the potential to disrupt the function of the affected genes (where the P-element was inserted in the opposite direction) for 47 EP and EPgy2 lines that have no obvious overexpressing lines. In short, a total of 1,183 lines were screened. 85 modifiers comprising 71 suppressors and 14 enhancers were identified in this primary screen. The 7% calculated hit rate is slightly higher than the 1–4% typically observed in similar screens, demonstrating the success of the screen in identifying modifiers. To exclude possible false positive, the modifying effect of these mutant lines was validated by using an independent allele and an additional assay.

Pan-neural expression of DVAP-P58S using the *elav-GAL4* driver causes lethality at 30°C. However, about 70% of the offspring survive when these flies are raised at 28°C. Survivors exhibit a normal locomotion behavior upon eclosion but 4 d later, they experience a progressive decline in climbing ability and at day 10 they exhibit severe incoordination and eventually, paralysis. Controls do not display any alterations in motor performance over the entire time period of the assay.

Interestingly, we found that the large majority of modifiers affect both disease phenotypes in the same direction and overall, there was a strong correlation between the degree of the modifying effect on the neurodegeneration and on the locomotion defects, suggesting that these 2 phenotypic readouts effectively converge in identifying genes belonging to the DVAP interactome.

## Computational Analysis of Genetic Data

Although our screen covered less than 10% of the *Drosophila* genome, we identified a fairly high number of modifiers by screening collections of available mutant lines. Over the past few years, advances in bioinformatics have provided researchers with powerful analysis tools that are of invaluable importance in interpreting large sets of experimental data and in expanding their significance.

A first step in this analysis is the ability to organize the identified modifiers in functional categories and to determine those functional categories that are overrepresented within the set of phenotype modifying genes. Ranking the genetic modifiers into a list of enriched functional categories can help in understanding the relative importance of specific cellular processes for the disease pathogenesis while the clustering of several modifiers within the same category validates the process as being important for the disease. Additionally, accurate and extensive computational analysis of large data sets of genetic data can inform and guide further experimental investigations. Hence, we undertook bioinformatic approaches and we performed an extensive computational analysis of modifier genes in order to extrapolate cellular processes and to define the network of interacting genes underlying ALS pathogenesis.

The 85 modifier genes were analyzed using the R/Bioconductor package ‘topGO’^36^ and this analysis revealed that a variety of cellular processes may be involved in ALS pathogenesis. Among them, topGO identified endosomal regulation, lipid particles, vesicular trafficking and cell proliferation/apoptosis as the most enriched functional categories. To further extend our analyses we searched the GeneMania database to determine whether the list of identified *Drosophila* modifiers can be connected into an interactome based on physical and genetic interactions as well as co-localization studies.^37^ Out of the 85 genetic modifiers identified, 72 were found to interconnect as a complex interactome, while previous studies have identified 7 of the modifiers (CG7324, Syx7, Ero1L, Rab5, CG5118, rho and Spp) as DVAP binding partners. Collectively, these data confirm and expand cellular processes and molecular pathways associated with DVAP functions and provide insights into possible molecular mechanisms underlying ALS8 pathogenesis *in vivo*.

## Relevance of the *Drosophila* Data for the Human Disease

Perhaps the main and ultimate reason for undertaking genetic modifier screens in *Drosophila* is the ability to identify the human orthologues of the fly modifier genes that may represent risk factors or be causative of the human disease.

We used DIOPT, a recently developed tool for ortholog mapping,^38^ and we found that out of the 85 modifiers 77 have a human homolog. The human genes were subjected to analysis with the DIST tool on DIOPT (http://www.flyrnai.org/diopt-dist) to determine whether any of them is known to be associated with any neurological disorder in humans. Information retrieved from the Online Mendelian Inheritance in Man data sets and Genome-Wide Association Studies indicates that a considerable number of human homologues of the fly modifiers is associated with neurodegenerative diseases such as Parkinson and Alzheimer diseases as well as spinocerebellar ataxias and multiple sclerosis. Other human orthologues were linked to psychiatric disorders such as schizophrenia, mental retardation and autism spectrum disorder. Although these data need to await experimental validation, they indicate that molecular commonalities exist between ALS8 and a broad range of neurological disorders.

As for the *Drosophila* genes, to determine whether the human orthologues of the *DVAP-P58S* modifiers could be linked into a molecular network of interacting genes, we turned to the Ingenuity Pathway Analysis (IPA database, Ingenuity system at www.ingenuity.com) database that integrates a variety of experimental sources including proteomic studies to establish relationships between human genes. The resulting human interactome included hVAPB and 31 additional genes out of the 77 human homologues. More importantly, these genes are also components of the *Drosophila* genetic network showing that a significant overlap exists between the fly and the human interactomes, confirming the relevance of the fly data for the disease pathogenesis in humans. To expand the network of the human interacting genes, we searched the IPA database for genes interacting with hVAPB and we included these genes in the list of the 77 human orthologues. By doing this, we were able to expand the network of human interacting genes and include 12 additional human genes homologous to the *Drosophila* modifiers that were not inserted in the first interactome.

To analyze the *in vivo* relevance of the computational data, we focused on a sub-network of computationally predicted interactors that includes *Drosophila* proteins implicated in endocytosis and vesicular trafficking based on GeneMania data base. A similar network was generated for the human orthologues of these genes using the IPA database. One of the gene that is part of both networks is Rab7, a marker for late endosome that was not identified by the screen, although upregulation of the early endosome marker Rab5 was found to be a potent suppressor of the disease phenotypes in flies. Interestingly, overexpression or constitutive expression of Rab7 was also found to be a potent suppressor of both the neurodegenerative and the locomotion defect phenotypes confirming the *in vivo* relevance of computationally derived interactions. Furthermore, Rab5 abnormally accumulates in the DVAP-positive inclusions in eye imaginal discs and brains expressing the pathogenic transgene. A similar redistribution of Rab5 was observed in *post-mortem* spinal cord tissues of ALS patients where Rab5 immuno-reactivity was found to form clusters and to partially co-localize with hVAPB aggregates. Interestingly, we noticed that, in controls, hVAPB immunoreactivity was restricted to large motor neurons where it appears as a granular staining diffused throughout these large cells.

Among the computationally predicted interactors there were also SOD1 and the causative gene of Huntington's disease huntingtin. Further experiments need to be performed to validate the *in vivo* relevance of these novel and potentially interesting interactions.

**Figure 2.**
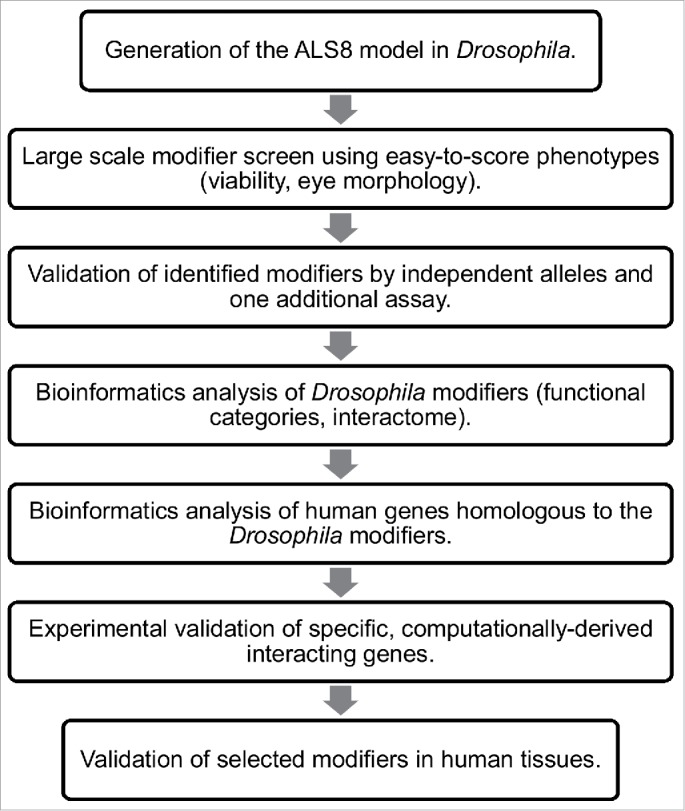
Flowchart of the screen for genetic modifiers of ALS8. The screening process in *Drosophila* followed by computational analysis of modifiers both in *Drosophila* and in humans and validation of a specific subset of modifiers in human tissues are depicted.

In summary, we show that computational analysis can help to understand but also to expand the significance of large data sets derived from genetic modifier screens in *Drosophila*. A careful analysis of computationally predicted interactomes may also guide further experimental investigation (**Fig. 2**).

## Repurposing the ALS8 Model

The *DVAP-P58S* modifier screen led to the identification of genes that were known to function in the disease pathogenesis or to be interacting partners of VAP proteins. As an example, a strong modifier was found to be the gene rdgBβ, a phosphoinositide phosphatase that has not been extensively characterized so far but is supposed to function in the same way as its closely associated isoform rdgB. RdgB is the homolog of Nir2 in humans that has been reported to interact with VAPB to control Golgi trafficking in human cell lines.^39^ A significant number of modifiers were genes involved in autophagy and the ubiquitin/proteasome system further supporting the role of protein clearance in ALS pathogenesis.

In an unbiased modifier screen however, one would wish to identify novel and so far unrecognized, processes as crucial contributors of disease pathogenesis. We were surprised to find that a number of genes controlling lipid droplet (LD) biogenesis and dynamics function as potent suppressors of the ALS8 disease phenotypes. LDs are organelles devoted to the storage and supply of neutral lipids. Although it is becoming increasing clear that LDs play a fundamental role in processes other than fat storage and mobilization,^40^ we do not know at the moment how alterations in LD biology could affect neuronal function and survival in ALS. However, previous proteomic studies have reported that DVAP is a protein associated with LDs^41^ and during starvation, abnormal clusters of LDs are observed in striated muscles of both *C. elegans* and mice in which the DVAP homolog gene has been inactivated.^42^ Among the identified modifiers, there is a striking enrichment for genetic pathways controlling cell proliferation and apoptosis such as the Ras pathway and the Hippo (Hpo) tumor suppressor pathway. Remarkably, we reported that *DVAP-P58S*-induced ALS phenotypes were sensitive to the dosage of a number of genes within the Ras pathway that function upstream of Raf and downstream of Ras including connector enhancer of KSR (CNK), Src42 and 14–3–3ζ.

Interestingly, reducing the expression levels of Hpo, a tumor suppressor gene, using 2 independently generated alleles suppresses both ALS8 phenotypes, the neurodegeneration and locomotion defects. This suggests that Hpo is likely up-regulated in neurons expressing the *DVAP-P58S* transgene. Multiple lines of evidence converge to support the role of Hpo in ALS8 pathogenesis. Firstly, Hpo has been reported to promote apoptosis by reducing the expression levels of a number of downstream targets including *Drosophila* inhibitor of apoptosis 1 (DIAP1).^43^ In fact, in *DVAP-P58S* mutant background, DIAP1 is downregulated while *DVAP-P58S*-induced neurodegeneration is alleviated by an up-regulation of DIAP1.^16^ Secondly, Hpo overexpression represses cell proliferation and induces apoptosis^44^ and crucially, its mammalian homolog MST1 is hyperactivated in *post-mortem* tissues of ALS patients while its down-regulation attenuates disease symptoms in SOD1 mouse models of ALS.^45^ Lastly, DVAP was identified as a high-confidence member of the Hpo interactome in high throughput protein-protein interaction analyses searching for additional components of the Hpo pathway.^46^

It is important to underline that genetic screens can also lead to the identification of potential targets for therapeutic intervention. This is indeed the case for Hpo since compounds acting as inhibitors of Hpo activity and therefore with the potential to suppress the ALS8 disease phenotypes, are available.^47^

The contribution of this *Drosophila* model to the discovery process of therapeutics for ALS8 however, may go beyond the simple identification of a potential therapeutic target as it can also be used as a platform for low to high throughput drug screening. Traditional approaches in high throughput drug screening of chemical compounds rely on studies in cell culture systems and on biochemical assay. Most positive hits identified through these types of *in vitro* assays turn out to be ineffective or toxic in subsequent validation experiments using rodent animal models.^48^ Ideally, one would like to perform primary drug screenings of large collections of compounds directly in whole animals but traditional murine models cannot be used because of time and highly prohibitive costs of the experiments. *Drosophila* is emerging as an excellent platform for medium to high throughout screenings of thousands of molecules that can quickly identify a number of high quality lead compounds. This approach depends on fully or partially automated scoring of visible phenotypes including eye morphological phenotypes, viability and simple behavioral assays such as climbing activity. The *Drosophila* ALS8 model we present here exhibits all of the features that would be required for such application.

While preparing this manuscript, 2 large-scale modifier screens for *C9orf72*-related ALS using the eye neurodegenerative phenotype as a phenotypic readout led to the identification of nucleocytoplasmic transport as a crucial contributor of ALS pathogenesis. *Drosophila* data were confirmed in human mammalian cells lines and in induced pluripotent stem cells derived from ALS patients affected by a *C9orf72*-linked ALS. These results obtained using other *Drosophila* models for ALS confirm and reinforce the role of *Drosophila* in identifying and elucidating fundamental aspects of ALS pathogenesis relevant for the human disease.^49,50^ These data also indicate that morphological alterations in the adult eye due to the targeted expression of disease causative genes, represent for ALS as for other neurodegenerative diseases, a powerful phenotypic readout in large scale screens for genetic modifiers.

Despite these results, one could still wonder how a smaller and rough eye phenotype in flies could be compared with the pathological features of complex human diseases such as ALS.

The recently emerged concept of “phenologs” may revolutionize the way we conceive a model of a human disease. It is becoming increasingly clear that genes are not conserved during evolution as independent entities but rather as a set of interacting genes that can be repurposed to control divergent processes in different species.^51^ Recent studies have shown that divergent processes such as angiogenesis in mutant mice and growth rate of yeast exposed to the hypercholesterolemia drug lovastatin share a large number of orthologous interacting genes so that yeast can be used as a model for angiogenesis in mammals.^51^ These two phenotypes are said to be “phenologs” since they present a significant overlap in the network of interacting genes despite the difference at the organismal level in phenotypic outcomes between the 2 species. Other examples of “phenologs” have been described and this discovery has led to the possibility that non-obvious models may be used to identify previously unrecognized genes important for a specific human disease.

In summary, it appears that *Drosophila* models of human neurodegenerative diseases can have a variety of applications. They can be used to dissect the molecular mechanisms underlying disease pathogenesis that may also lead to the identification of specific therapeutic targets. Models can provide a valuable platform for screening large libraries of chemical compounds, if suitable phenotypic readouts are available. Large scale and unbiased genetic screens for modifiers of *Drosophila* disease phenotypes combined with bioinformatic analysis of the identified hits can inform scientists about the fundamental aspects of disease pathogenesis in humans and guide further experimental investigations (**Fig. 2**). Finally, the experimental strategy we describe here provides a pool of candidate genes that could inform Genome-Wide Association Studies in humans leading to an effective increase in the rate of discovery of new disease causative genes.
